# Keeping the Customer Satisfied: Applying a Kano Model to Improve Vaccine Promotion in the Philippines

**DOI:** 10.9745/GHSP-D-23-00199

**Published:** 2023-12-22

**Authors:** Jonas Wachinger, Mark Donald C. Reñosa, Jerric Rhazel Guevarra, Jhoys Landicho-Guevarra, Maria Paz Demonteverde, Catherine Silvestre, Vivienne Endoma, Jeniffer Landicho, Mila F. Aligato, Thea Andrea Bravo, Rachel P. Chase, Shannon A. McMahon

**Affiliations:** a Heidelberg Institute of Global Health, Heidelberg University Hospital, Heidelberg, Germany.; b Department of Epidemiology and Biostatistics, Research Institute for Tropical Medicine – Department of Health, Muntinlupa, Philippines.; c Department of Research Information Technology, The Ohio State University College of Medicine, Columbus, OH, USA.; d Department of International Health, Johns Hopkins University Bloomberg School of Public Health, Baltimore, MD, USA.

## Abstract

**Introduction:**

The success of global health interventions heavily relies on reaching populations in a way that aligns with their priorities and needs. This warrants novel approaches to determine the design of meaningful interventions and targeted delivery pathways. To date, global health scholarship and practice have largely underused approaches already established in fields that emphasize customer satisfaction, such as quality management or consumer psychology.

**Methods:**

In our study, we apply Kano methodology—originally designed to understand how product attributes nonlinearly influence customer satisfaction—to inform design decisions regarding a video-based vaccine intervention in the Philippines. Between September 2021 and April 2022, we administered a Kano questionnaire to 205 caregivers of small children. Data were analyzed following routine Kano approaches, supplemented by cultural consensus analysis (CCA), which is an approach used largely in anthropology to identify distinct cultural groups and competencies.

**Results:**

Applying Kano and CCA methodologies allowed us to make informed design decisions in terms of optimizing accessibility and credibility of an intervention that ultimately proved successful in bolstering vaccine intentions. Results guided us to include national and international logos, to appreciate the value of summarizing key messages, and to recognize the importance of fact- or story-based communication as attributes that influenced respondent satisfaction one-dimensionally. We found that involving trusted messengers and including text-based information were required to avoid dissatisfaction. Interacting with someone after viewing the product and creating opportunities to share the promotional material via social media were attractive attributes whose presence would increase satisfaction but would not spark severe dissatisfaction if omitted. Other attributes (short duration, video- or animation-based intervention, delivering the intervention at health centers or in group settings) played a limited role in respondent satisfaction.

**Conclusions:**

Global health research and practice can benefit from applying approaches established in other fields when making evidence-based prioritization decisions to tailor interventions.

## Abstract (precis)

The authors show how global health science and practice can benefit from applying approaches established in other fields, such as consumer psychology and quality management, to increase clients' satisfaction with health interventions.

## Teaser

**Key Findings:**

- Approaches outside the established global health repertoire, such as Kano methodology or cultural consensus analysis, can open novel and resource-friendly pathways for the science and practice of developing and implementing health interventions.
- In our study, intervention attributes related to increasing accessibility (e.g., subtitles, content summaries, social media shareability) and credibility (e.g., including logos, involving trusted messengers, allowing for in-person interaction) were most relevant for respondent satisfaction.
- We describe how we translated Kano evidence into practice and recommend considering contextual factors, for example, by integrating attractive attributes to give the developed intervention a “competitive edge” over conflicting health messaging or by exploring combinations of attributes that can coexist in a given setting.

**Key Implications:**

- Intervention designers and implementers should consider approaches such as Kano and cultural consensus analysis when aiming to align global health interventions with the needs and priorities of populations.
- Global health researchers can explore methodologies outside the standard global health repertoire when seeking novel pathways to design health interventions.

## INTRODUCTION

For decades, scholars have highlighted how global health communication can draw on the breadth of experience available in consumer research, marketing psychology, and related fields when aligning intervention design and implementation with populations' needs and priorities.^1^ However, to date, many promising approaches established in these disciplines remain underused in the global health context. One such approach that has been found to be promising in the private sector to facilitate the identification and prioritization of different product attributes and their impact on client satisfaction is the Kano method.^2^

**FIGURE 1:**
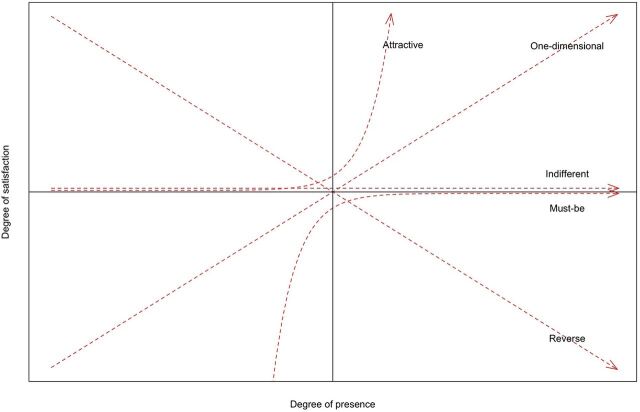
Relationships Between Types of Quality Attributes and End-User Satisfaction in the Kano Model

As opposed to the intuitive assumption of a linear relationship between a given quality attribute of a product and end-user satisfaction (i.e., the more any attribute is present, the more satisfied a product's end users are), the Kano model proposes a typology of quality attributes where most attribute types influence end-user satisfaction nonlinearly.^3^ These attribute types are called must-be (M), attractive (A), one-dimensional (O), reverse (R), and indifferent (I) attributes. Additionally, classifications can fall in the questionable (Q) category (Table 1).^2^^,^^3^ The Kano model posits that each quality attribute type has a distinct pattern of influencing end-user satisfaction as the presence of the respective quality attribute in the product increases or decreases. Figure 1 shows a standard visualization of the patterns; questionable attributes do not have a consistent and plottable relationship between presence and satisfaction. In addition to the terms “presence” versus “absence” to distinguish between levels of attribute performance, authors have also used “functional” versus “dysfunctional,”^4^ or “sufficient” versus “insufficient.”^5^ In this article, we will use the “presence” versus “absence” terminology while acknowledging that “presence” in many cases is not dichotomous but that the level of perceived attribute presence might be shaped by an end user's perception of attribute performance.

**TABLE 1.** The 6 Types of Attribute Classifications in the Kano Model

Quality Attribute	Definition
Must-be (M)	Basic requirements of a product: Absence of must-be attributes leads to increased dissatisfaction, but their presence is not associated with increased satisfaction.
Attractive (A)	Cherries on top: Absence of attractive attributes does not cause dissatisfaction, but their presence increases satisfaction considerably.
One-dimensional (O)	The more the better: Satisfaction increases linearly with the degree to which a one-dimensional attribute is present.
Reverse (R)	Presence of reverse attributes causes dissatisfaction, but their absence is appreciated.
Indifferent (I)	Whether present or absent, indifferent attributes don't lead to an increase in satisfaction or dissatisfaction.
Questionable (Q)	Response patterns to the Kano questionnaire are contradictory (e.g., when a respondent indicates that an attribute must be present and must not be present at the same time).

Since the Kano model was first proposed, studies have provided further evidence to support this nonlinear understanding of the relationship between attribute presence and end-user satisfaction.^5^^,^^6^ Consequently, the model has received substantial attention and has been applied to a broad spectrum of contexts, including the tourism industry,^7^ when evaluating NASA's microgravity science program,^8^ designing skiing equipment,^9^ or for understanding preferred characteristics of employees dealing with complaining customers.^10^

Understanding how to classify product quality attributes in terms of the Kano model allows researchers, product designers, and implementers to identify the impact of various product attributes on end-user satisfaction and thereby prioritize specific attributes in the development and roll-out process.^4^ A standard interpretation of the model is that attributes identified as being mainly indifferent can be postponed or disregarded in product development, but must-be attributes should generally receive highest prioritization, followed by one-dimensional and attractive attributes.^11^^,^^12^ However, in Materla and colleagues' systematic review, the authors called for making Kano-guided development prioritization decisions with contextual factors in mind, for example, in situations when prioritizing attractive attributes might give a company considerable advantage over a competitor's product or when the timely achievability of attributes differs.^3^

In the health care context, authors have highlighted the potential of the Kano model, especially in light of clients' role changing from passive recipients of care to cocreators of the care delivery approach.^3^ Despite this potential and increasing momentum in recent years,^3^ applications of the Kano approach remain limited in the health field, with studies focusing largely on health care service delivery in various settings, including Greece,^13^ Sweden,^14^ Brazil,^15^ and Peru.^16^ More recently, the Kano approach has informed assessments of mHealth technologies,^17^ teleconsulting applications,^18^ and intelligent medication administration systems.^19^

However, we are not aware of studies investigating the potential of the Kano model to inform research on or the practice of developing novel global health communication interventions. In this study, we aim to contribute to filling this gap as we use and expand on the Kano approach to identify the roles of different quality attributes of vaccine confidence messaging in the Philippines and outline how these insights can meaningfully inform design practice.

## METHODS

### Study Setting

The Philippines had been a country with high vaccine confidence and vaccination rates^20^ before a highly publicized 2017 vaccine controversy after the introduction of a novel dengue vaccine resulted in plummeting vaccination rates and a rapid decline in vaccine confidence across the country.^21^ This study is part of a larger project that draws on human-centered design to codevelop an empathy-based vaccine confidence intervention together with end users in the Philippines.^22^^,^^23^ Qualitative findings from this project highlight how the intended population experienced barriers to vaccination uptake, including structural factors and discouraging experiences when accessing health care^24^ and how intrahousehold dynamics shape the vaccination decision-making process,^25^ providing further detail on intervention context and end users.

### Questionnaire Design

To assess the quality attributes of vaccine confidence messaging in this setting, we developed a questionnaire following the Kano approach. Each respondent was asked 2 questions per quality attribute to be assessed: 1 on their reaction if this attribute was present in the final product (functional question) and 1 on their reaction if it was not (dysfunctional question).^4^ For each question, the respondent could choose 1 of 5 response options (Table 2).

**TABLE 2.** Functional and Dysfunctional Questions and Their Response Options

Question	Response Options
Functional question: How would you feel if attribute X were present in the final product?	(a) I like it that way. (b) It must be that way. (c) I am neutral. (d) I can live with it that way. (e) I dislike it that way.
Dysfunctional question: How would you feel if attribute X were not present in the final product?	

To identify the quality attributes relevant to our study, we drew on qualitative data collected in previous phases of the overarching project,^22^ as well as on health communication literature. Based on iterative discussion within the study team, 14 attributes were ultimately selected as being relevant quality attributes of vaccine confidence communication in the context of our study (Table 3). Additionally, respondents were asked questions about their sociodemographic profile, vaccination attitudes, past vaccination behavior, and sources of vaccine-related information.

**TABLE 3.** Attributes Included in the Kano Questionnaire and Their Descriptions

ID	Attribute	Description
	Intervention characteristics	
A1	Video	Preference to receive vaccine information as a video
A2	Cartoon	Preference to receive vaccine information in animated form
A3	Short duration	Preference for a short communication product (less than 5 minutes)
A4	Text	Preference for a video-based intervention to include written text, such as subtitles
A5	Resource information	Preference to receive a pamphlet after the intervention which highlights further resources and lists contact addresses
A6	Key message summaries	Preference to receive a pamphlet which summarizes the key messages of the communication product
	Intervention distribution	
B1	Shareability	Preference to be able to share the communication product via Facebook or other social media
B2	Trusted messenger	Preference for the communication product to be distributed by a known and trusted person
B3	Health center location	Preference for the communication product to be screened/distributed at the health center
B4	Privacy	Preference to be alone when watching the communication product
B5	National logo	Preference for the communication product to include the logos of national organizations and authorities
B6	International logo	Preference for the communication product to include the logos of international organizations and authorities
	Intervention content	
C1	Fact-based	Preference for the product to focus on presenting scientific facts
C2	Story-based	Preference for the product to include narratives and stories of individuals

### Sampling and Data Collection Procedures

With the support of health workers, we recruited caregivers of children aged younger than 5 years in 2 rural and 2 urban barangays (the smallest administrative unit in the Philippines). Because participants may require some explanation or guidance when first encountering a Kano questionnaire, oral interviews have been argued to be the most suitable approach for Kano data collection.^11^ Due to the prolonged COVID-associated lockdown at the time of data collection (September 2021 to April 2022), data collection was conducted via video call on the Facebook Messenger platform (as preferred by participants) in the form of interviewer-assisted surveys; mobile data packages were transferred to participants to account for study-associated data usage. All participants provided online informed consent before their participation.

### Data Analysis

First, we analyzed Kano questionnaire data using the original Kano approach^2^ by assigning each individual pair of answers per attribute to 1 of the 6 categories of quality attributes (Table 4), followed by computing the total number of responses falling in each category. Each individual attribute was then classified based on the modal value of individual classifications.^12^ For the privacy attribute (B4)—watching the communication product when alone—a majority of responses fell into the reverse (R) category. We followed the standard approach for coding such attributes by treating the functional question as dysfunctional and vice versa, thereby computing an attribute indicating the preference for watching the product in the presence of others (resulting in the new attribute B4R).

**TABLE 4.** Classification Assignment in the Kano Model^a^

	Dysfunctional Question (Attribute Not Present)				
	1: Like	2: Must-Be	3: Neutral	4: Live With	5: Dislike
**Functional question (Attribute present)**					
**1: Like**	Q	A	A	A	O
**2: Must-Be**	R	I	I	I	M
**3: Neutral**	R	I	I	I	M
**4: Live With**	R	I	I	I	M
**5: Dislike**	R	R	R	R	Q

Abbreviations: A, attractive; I, indifferent; M, must-be; O, one-dimensional; Q, questionable; R, reverse.

a For example, if a respondent answers the functional question with “I like it that way” and the corresponding dysfunctional question with “I dislike it that way,” then the respective attribute is classified as one-dimensional.

Second, as response patterns in our data proved to be highly diverse, with 2 or more categories often close to being tied, we followed Blauth and colleagues' recommendation for such cases.^26^ In addition to the traditional Kano analysis, we assessed whether the sum of high-relevancy categorizations—must-be (M), attractive (A), and one-dimensional (O)—for a given attribute outweighed the low-relevancy categorizations—indifferent (I), reverse (R), and questionable (Q). If this was the case, we assigned the high-relevancy category that had received the most responses following the formula^26^: if (M+O+A)>(I+R+Q), then grade is max(M; O; A), else grade is max(I; R; Q).

Additionally, given the high variation of classifications within attributes, we conducted a cultural consensus analysis (CCA)^27^ to assess whether our sample consisted of more than 1 consensus pattern that we could identify as “market segments.”^4^ If the CCA indicates the existence of more than 1 group, each with distinct response patterns, further investigation of the individuals expressing those different consensus patterns could reveal if those groups differed in other ways we had measured, for example, according to certain sociodemographic characteristics or past vaccination behavior. Additionally, CCA allows for estimating “culturally true” responses to questions based on weighing individual respondents' answers according to their estimated competency within their CCA-identified group. We drew on a general condorcet model for binary data, using the dichotomization approach previously outlined (M, O, A=1; I, R, Q=0) to assess whether a given attribute was likely relevant for satisfaction in the group represented in our sample. Additionally, we calculated eigenvalues to create a scree plot using data dichotomized following M, O, A, R=1; I, Q=0 to account for potential underestimation of individuals assessing a given attribute as relevant for satisfaction when treating reverse cases as irrelevant categorizations.

Finally, we computed importance coefficients as recommended by Timko,^28^ which have since become a standard supplement to the traditional Kano analysis. In this approach, 2 coefficients are calculated that highlight how a given attribute affects end-user satisfaction if it is more present (the “[if we perform] better” coefficient) and if it is more absent (the “[if we perform] worse” coefficient). For this, the following formulas are being applied^28^:

$$\mathrm{Better}=\frac{A+O}{A+O+M+I}\mathrm{Worse}=\frac{O+M}{A+O+M+I}$$

Both coefficients range from 0 to 1 and highlight how end-user satisfaction increases if we provide this attribute and how satisfaction decreases if we fail to provide this attribute, respectively.^28^ For example, if all clients classify an attribute as attractive, then better=1 and worse=0, which signifies that if product designers perform better at providing this attribute, satisfaction will increase, but satisfaction will not decrease if this attribute is not provided. The resulting 2 coefficients for each attribute can then be plotted in a graph, allowing visualization of their impact both on satisfaction and dissatisfaction, as well as relative to other attributes (this exemplary case would be plotted at 0,1 in the top left, attractive corner of the graph).^28^

All data were analyzed using RStudio (4.2.2), using the CCTpack (1.5.2) package for CCA (samples=10,000, chains=3, burnin=2,000, measuring and accounting for item difficulty and calculating posterior predictive checks).

### Ethical Approval

This study received ethical approval from the institutional review boards at the medical faculty of Heidelberg University, Germany (S-833/2019) and the Research Institute for Tropical Medicine, the Philippines (2019-44). All methods were performed in accordance with the relevant guidelines and regulations, including the Declaration of Helsinki and the Belmont Report.

## RESULTS

### Demographic Characteristics

Our sample (n=205) consisted predominantly of women (n=200, 98%), and respondents, on average, were aged 32 years (interquartile range 27 years to 38 years) (Table 5). Thirty-one respondents (15%) described not having received vaccine information previously. The families of 7 respondents (3%) were part of the Pantawid Pamilyang Pilipino Program, commonly known as 4Ps,^29^ which allows lowest-income families to obtain certain welfare benefits if they, among other requirements, adhere to child vaccination and health checkup goals.

**TABLE 5.** Sociodemographic Characteristics of the Study Sample

Characteristics	No. (%) (N=205)
Gender	
Female	200 (98)
Male	5 (2)
Educational background	
Never attended school	2 (1)
Elementary	22 (11)
High school undergraduate	26 (13)
High school graduate	90 (44)
Vocational education	9 (4)
College undergraduate	32 (16)
College graduate	23 (11)
Graduate studies	1 (<1)
Occupation	
Housewife	106 (52)
None	32 (16)
Business (e.g., service and sales workers)	19 (9)
Self-employed (e.g., online business, retail)	16 (8)
Professional (e.g., doctors, engineers, IT)	13 (6)
Manual laborer (e.g., craft and related trade workers)	12 (6)
Farmer	0 (0)
Fisherman	0 (0)
Others	7 (3)
Vaccination behavior	
Previously delayed or refused vaccination	56 (27)
Previously delayed vaccination	41 (20)
Previously refused vaccination	21 (10)
Would vaccinate their child today	196 (96)
Previous sources of vaccination information (multiple answers possible)	
Barangay health workers	104 (51)
Family members	69 (34)
Midwives	48 (23)
Social media	39 (19)
Television	33 (16)
Nurses	27 (13)
Friends	22 (11)
Doctors	11 (5)
Newspapers	10 (5)

Abbreviation: IT, information technology.

### Kano Analysis and CCA

The CCA scree plot (Supplement) suggested that our sample represents 1 cohesive group; 2 cases with no variance were omitted when calculating the respondent-respondent correlation matrix. A similar pattern emerged even if reverse classifications were treated as relevant (Supplement). Mean respondent competency and mean guessing bias were both estimated at 0.34 (standard deviation=0.14 and 0.172, respectively).

Table 6 outlines the classifications per attribute, as well as the overall classification via the traditional Kano model (column “Resulting Classification, Original”), Blauth and colleagues' formula (column “Resulting Classification, Revised”), as well as the CCA-based classification via the following formula in column “Resulting Classification, CCA”: If item_truth>0.5 then grade is max(M; O; A), else grade is max(I; R; Q).

**TABLE 6.** Kano Classifications and CCA Results

	Individuals Classifying Attribute as A/O/M/I/R/Q Type, %						Resulting Classification			Estimated Binary Truth Value	CCA Item Truth	Item Difficulty
Attribute	A	O	M	I	R	Q	Original	Revised	CCA			
A1 video	31	6	4	38^a^	11	11	I	I	I	0	0.02	0.74
A2 cartoon	13	1	8	42^a^	28	8	I	I	I	0	0.00	0.30
A3 short duration	6	2	12	52^a^	22	5	I	I	I	0	0.00	0.24
A4 text	24	20	26^a^	25	3	0	M	M	M	1	1.00	0.42
A5 resource information	29^a^	21	18	29	1	1	A	A	A	1	1.00	0.48
A6 key message summaries	22	23	20	33^a^	1	1	I^b^	O^b^	O^b^	1	1.00	0.53
B1 shareability	27	20	17	30^a^	5	2	I^b^	A^b^	A^b^	1	1.00	0.59
B2 trusted messenger	11	12	27	36^a^	4	10	I^b^	M^b^	M^b^	1	0.69	0.88
B3 location	29	13	9	31^a^	2	16	I^b^	A^b^	I^b^	1	0.45	0.88
B4R company	23	9	7	38^a^	10	13	I	I	I	0	0.01	0.72
B5 national logo	19	40^a^	19	20	1	0	O	O	O	1	1.00	0.25
B6 international logo	18	24	22	34^a^	1	1	I^b^	O^b^	O^b^	1	1.00	0.54
C1 fact-based	10	57^a^	22	11	0	0	O	O	O	1	1.00	0.09
C2 story-based	13	30^a^	29	26	1	1	O	O	O	1	1.00	0.40

Abbreviations: A, attractive; CCA, cultural consensus analysis; I, indifferent; M, must-be; O, one-dimensional; Q, questionable; R, reverse.

a Represents the modal classification.

b Instances where classification varied between different classification approaches.

We additionally report item difficulty and item truth as estimated in the CCA. Instances where the different approaches resulted in differences in attribute classification are indicated by superscript letter b. Following the CCA-supported classification, it was considered a must-be attribute for the communication product to include subtitles or other written text (A4) and to be delivered by a trusted or well-known messenger (B2). Including summaries of key messages (A6), national (B5) and international (B6) logos, and fact- (C1) and story-based (C2) communication related one-dimensionally to respondents' satisfaction. Finally, receiving contact information and resources for follow-up questions (A5) or shareability of the final product via social media or other channels (B1) was considered attractive. All other attributes did not have a decisive impact on the satisfaction of a majority of our respondents.

As an additional resilience analysis, we removed high-difficulty items with obscure truth values (A1, B2, B3, B4R) and reran the scree plot and CCA analysis. Overall outcomes were not affected: a single cohesive group was still identified (Supplement), and all remaining attributes were still classified into the same category.

**FIGURE 2:**
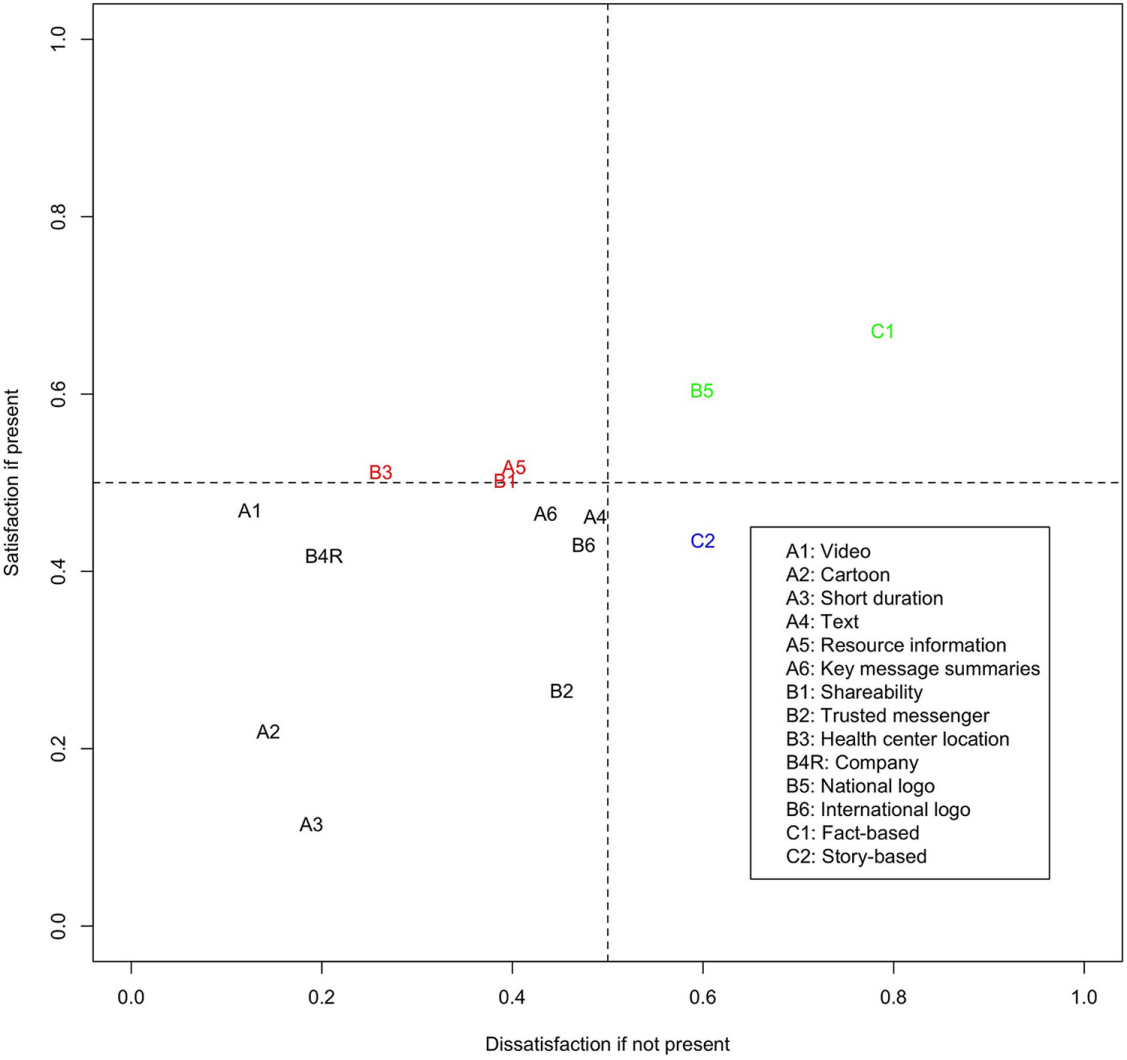
Mapped Kano Importance Coefficients

Figure 2 outlines the importance coefficients of all attributes, calculated based on Blauth and colleagues' formula. The x-axis highlights how much the absence of a particular attribute increases dissatisfaction; the y-axis indicates how much an attribute's presence sparks satisfaction. Attributes in the bottom right quadrant (C2: story-based) tend to be must-be attributes; attributes in the top right quadrant (B5: national logos and C1: fact-based) align rather with one-dimensional characteristics. The top left quadrant (A5: resource information, B1: shareability, and B3: location) indicates an alignment with attractive qualities, and respondents tend to be rather indifferent toward the remaining attributes in the bottom left quadrant.

## DISCUSSION

In this article, we transferred approaches established in the fields of quality management and consumer psychology to global health intervention research. We drew on the Kano model^2^ and CCA to outline how the presence or absence of different attributes of vaccine-promotive messaging related to clients' satisfaction with our vaccine confidence intervention. Delivering the intervention via a trusted messenger and including text (if video-based) were classified as must-be attributes, which, if omitted, would spark dissatisfaction. One-dimensional attributes included summaries of key messages, inclusion of national and international logos, and fact- or story-based communication. Receiving contact information or further resources and shareability of the communication product via social media were attractive attributes; other attributes were classified as mainly indifferent.

Applying CCA in addition to traditional Kano approaches allowed us to quantify the degree to which consensus existed regarding attribute relevance in our sample. Our CCA suggested that responses were best modeled as arising from a single cultural consensus rather than from 2 or more. Future research might employ stratified sampling to investigate further whether different consensus patterns exist in particular population subgroups not observed here. To the best of our knowledge, this is the first study to use CCA to assess whether separate Kano analyses should be applied to characterize market preferences of different consensus patterns within a broad population. We encourage future research that expands on these methods to better identify and understand differing and especially minority consensus patterns. This includes studies that stratify sampling to focus on sociodemographic characteristics or behavioral patterns that might predict or produce different consensus patterns, as well as studies that test whether employing this market research strategy improves outcomes over standard methods.

Our CCA results also indicated a rather low average and high variation in cultural competency, high variability in item difficulty, and high variability in how respondents classified each attribute. As a result, there are several attributes with clear outcomes (low-to-medium item difficulty<0.5 and consistent classification as being relevant across approaches), such as including text and national logos and fact- or story-based communication, which we prioritized as attributes to retain in the final product design. Attributes that were classified as indifferent (e.g., duration and audience environment) were subject to more scrutiny, with practical opportunities and limitations weighing more heavily on our final decisions. For several other attributes, particularly for those with higher item difficulty and conflicting classifications (e.g., messenger and location of delivery), our interpretation was heavily context dependent. For example, the delivery of the intervention at the health center did not elicit a clear classification pattern among respondents; therefore, we will consider this delivery option for initial implementation or pilot testing while continuing to explore alternative approaches (including via potential future Kano questionnaires comparing several approaches). For other attributes with ambivalent results (e.g., social media shareability or the inclusion of summaries), we will lean toward an inclusive approach, implementing these attributes where they would not require fundamental alteration or compromise other attributes. At the same time, for attributes being predominantly classified as indifferent but with a substantial share of reverse classifications (e.g., a cartoon-based intervention), we recommend a restrictive approach, implementing these attributes only under close monitoring and continuous evaluation.

In global health practice, some attributes might be combined rather easily in a final product, but others are mutually exclusive. In our setting, potential avenues for delivering the intervention (e.g., video screened at both the health center and in other settings) or certain content characteristics (e.g., including logos of both national and international organizations) might coexist. At the same time, heavily basing an intervention on both communicating scientific facts and on the lived experiences of individuals can be challenging. Therefore, we encourage public health intervention developers to base their attribute prioritization decisions not only on the overall classification of the respective attribute but also on their assessment of how the individual attribute relates to other intervention characteristics in their particular context.

The pattern in our data that different classification approaches can lead to conflicting classifications reflects previous scholarship. In their review of the first 30 years of Kano literature, Witell and colleagues highlighted how several different classification approaches for the Kano method have been proposed^30^ and that a systematic comparison has shown that these often lead to different results.^31^ Similarly, when suggesting the analytical approach of plotting continuous satisfaction and dissatisfaction values, Timko highlighted considerable statistical uncertainty and cautioned against using these values to differentiate between close results.^28^ This uncertainty is reflected in our data where attributes classified differently by the 2 approaches are clustered close to the 0.5 cut-off values. The discourse regarding how to strike a balance between the different approaches is ongoing and highlights a need for design and implementation researchers to not rely only on 1 seemingly objective classification but also consider the role of product and context when making design decisions.

Nevertheless, we see added value in the graphical continuous representation of results to account for responses often being spread across many different categories. In such cases, the graphical representation allows for a more differentiated interpretation^28^^,^^32^ and can inform attribute prioritization considerations. As a general approach for prioritization, Boger^32^ has recommended a counterclockwise movement, starting with attributes in the bottom-right corner (indicating maximum dissatisfaction if the attribute is not present). Although Boger's cascade reflects those of others who recommend first addressing must-be attributes, followed by one-dimensional, attractive, and then indifferent attributes,^11^ we also emphasize that prioritization decisions must be made with contextual factors in mind.^3^ In the context of public health interventions, we encourage designers and implementers to consider their aims and context. In some instances, a priority might be to ensure stakeholder buy-in based on preexisting expectations for the intervention (in which case a focus on must-be and one-dimensional attributes might be appropriate), but in other cases, an intervention might be competing with other products (e.g., in the context of conflicting health messaging) or aiming at exploring novel pathways to reach intended populations, in which case considering attractive attributes could give the intervention a “competitive edge.”

Several scholars have highlighted that attributes have “life cycles” and that classification of attributes can change as clients' perceptions and expectations develop.^30^^,^^33^^,^^34^ A substantive number of attributes in our study were classified as indifferent. Although this facilitates current prioritization of other attributes in the development process, we acknowledge scholarship that highlights how attributes can migrate from the indifferent category to the attractive category over time.^34^ In the longer term, we encourage researchers and implementers to not entirely disregard attributes currently classified as indifferent. Similarly, Kano^33^ argued that attribute life cycles commonly include transitions from attractive to one-dimensional to must-be. This suggests that some attributes that might have only gained traction in recent years (e.g., shareability via social media) may increase in importance in the future.

High variability in individual respondents' classifications of attributes is common and may indicate several overlapping market segments.^11^^,^^12^ Our approach of integrating CCA into Kano analysis allowed us to identify that respondents in our sample were best modeled as a single group. However, the observed variability in item difficulty (i.e., whether respondents agreed regarding an attribute being relevant for their satisfaction) highlights how not 1 shared cultural truth across all respondents might exist. For example, no clear shared truth emerged regarding the role of trusted messengers and the location of intervention delivery, which merits further investigation and outlines how CCA can meaningfully supplement established Kano analytical approaches. To the best of our knowledge, this is the first study combining the Kano model with cultural consensus theory. We thereby respond to previous criticism that Kano research has frequently been “more of the same,” with few studies moving beyond frequency counting to explore novel analytical avenues.^30^ We hope that employing cultural consensus theory might spark ideas for further development within the field.

Scholars employing the Kano method have highlighted differences depending on the group or cultural setting investigated. In the health care context, differences observed have included perceptions of Arab and Austrian patients,^35^ different nationalities' perceptions of hospital service quality in Thailand,^36^ or between patients and physicians in Spain.^37^ This aligns with experiences across design and implementation efforts in global health,^38^ and we would like to encourage researchers and implementers to be attentive to nuanced differences in their respective settings. Kano methodology might be 1 additional approach in the public health toolbox to make meaningful decisions regarding intervention design priorities.

### Limitations

Although we see the strength of our study in exploring the potential of the Kano method when designing novel health communication approaches, there are several points we would like to highlight for the consideration of future researchers to further improve quality and robustness. First, as several authors have highlighted, combining Kano-style questions with items regarding respondents' self-reported importance rating of each attribute (e.g., ranging from “not at all important” to “extremely important”) would further inform prioritization decisions by, for example, differentiating a must-be attribute whose absence would be a small nuisance from an attribute whose absence would make a product feel virtually useless.^11^^,^^12^^,^^30^ Additionally, scholars have noted that a high number of questionable classifications, which occurred for some attributes in our study, may suggest that some questions are hard to understand^11^ and merit rephrasing.^34^ Question understanding and the generalizability of our findings might also have been influenced by certain study characteristics, for example, that we collected our data via online calls during COVID-19-associated lockdowns. Although access to and familiarity with phones and online calls in the Philippines are generally high and our provision of data packages should have alleviated financial barriers to participation, this data collection approach might nevertheless have introduced sampling biases or contributed to potential misunderstandings regarding the specific Kano question format. Such potential biases might have affected the number of questionable classifications or the fact that, according to the CCA results, our respondents formed 1 cohesive group. In general, we encourage researchers to pilot-test their Kano questionnaire (e.g., by using cognitive interviewing) to rephrase questions, ensure clarity of response options, and assess other factors, such as how a data collection setting might affect results. Specifically related to our study, although our results were helpful in refining the ultimately successful^23^ intervention design, broad generalizations to other settings and populations before further validation of the applicability of our findings might merit caution. Finally, we echo previous calls^30^ to explore novel pathways that investigate how different attributes and their impact on client satisfaction can interact. Clients might initially be indifferent to a given attribute, but in the context of survey administration, they may revisit this classification. This within-survey change in attribute perception could be systematically investigated by, for example, routinely changing combinations and ordering of individual attributes in administered questionnaires.

## CONCLUSIONS

Approaches established in other fields can meaningfully inform global health research and practice. In the context of vaccine confidence interventions in the Philippines, our results highlight how Kano and CCA methods can outline how certain attributes that increase accessibility (location of delivery, including text or subtitles, and offering the possibility of interaction following video viewing) and trustworthiness (including logos of national and international organizations and employing trusted messengers) of a communication product could influence satisfaction beyond the content of the product (fact- and story-based) itself. Other attributes, such as the product's duration (more or less than 5 minutes) or whether it is in cartoon format, are not likely to have a striking influence on our study population. We encourage researchers and other stakeholders to consider these findings in their design and implementation efforts, both regarding the Filipino setting and when exploring pathways to acknowledge client feedback on the relevance of certain attributes of other interventions in different contexts.

Since being first described, the Kano model has had a considerable impact on the conceptualization of how product attributes can affect customer satisfaction, but research has traditionally been heavily focused on management and marketing fields.^30^ Although in recent years, literature on utilizing the Kano approach for improving health care has increased,^3^ its application in the context of global health interventions has remained limited. With our study, we hope to contribute to an increased awareness and practical utilization of the Kano model's potential in exploring the relevance of different attributes of novel health communication interventions for client satisfaction.

## Supplementary Material

23-00199-Wachinger-Supplement.pdf

## Notes

**Peer Reviewed**

**First published online:** December 6, 2023.
