# Supplementary material for: Keeping the Customer Satisfied: Applying a Kano Model to Improve Vaccine Promotion in the Philippines
Source: Glob Health Sci Pract. 2023 Dec 22;11(6):e2300199. doi: 10.9745/GHSP-D-23-00199 (PMC10749646; doi:10.9745/GHSP-D-23-00199)

Supplement. Cultural Consensus Analysis (CCA) – Scree plots

Figure S1: CCA scree plot based on all attributes, dichotomized following (M | O | A = 1, I | R | Q = 0), omitting two respondents with zero response variance.

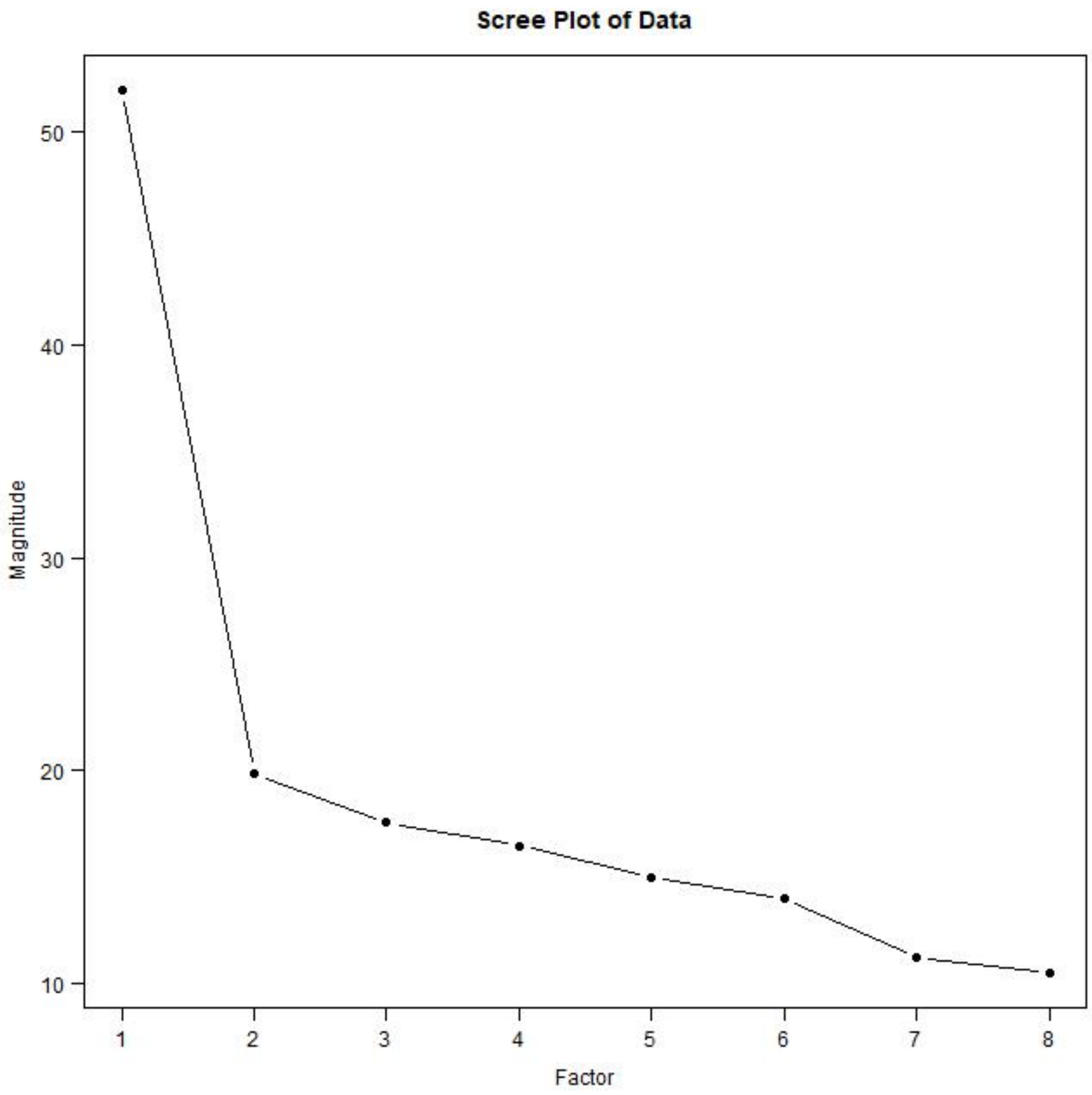

Figure S2: CCA scree plot based on all attributes, dichotomized following (M | O | A | R = 1, I | Q = 0), omitting three respondents with zero response variance.

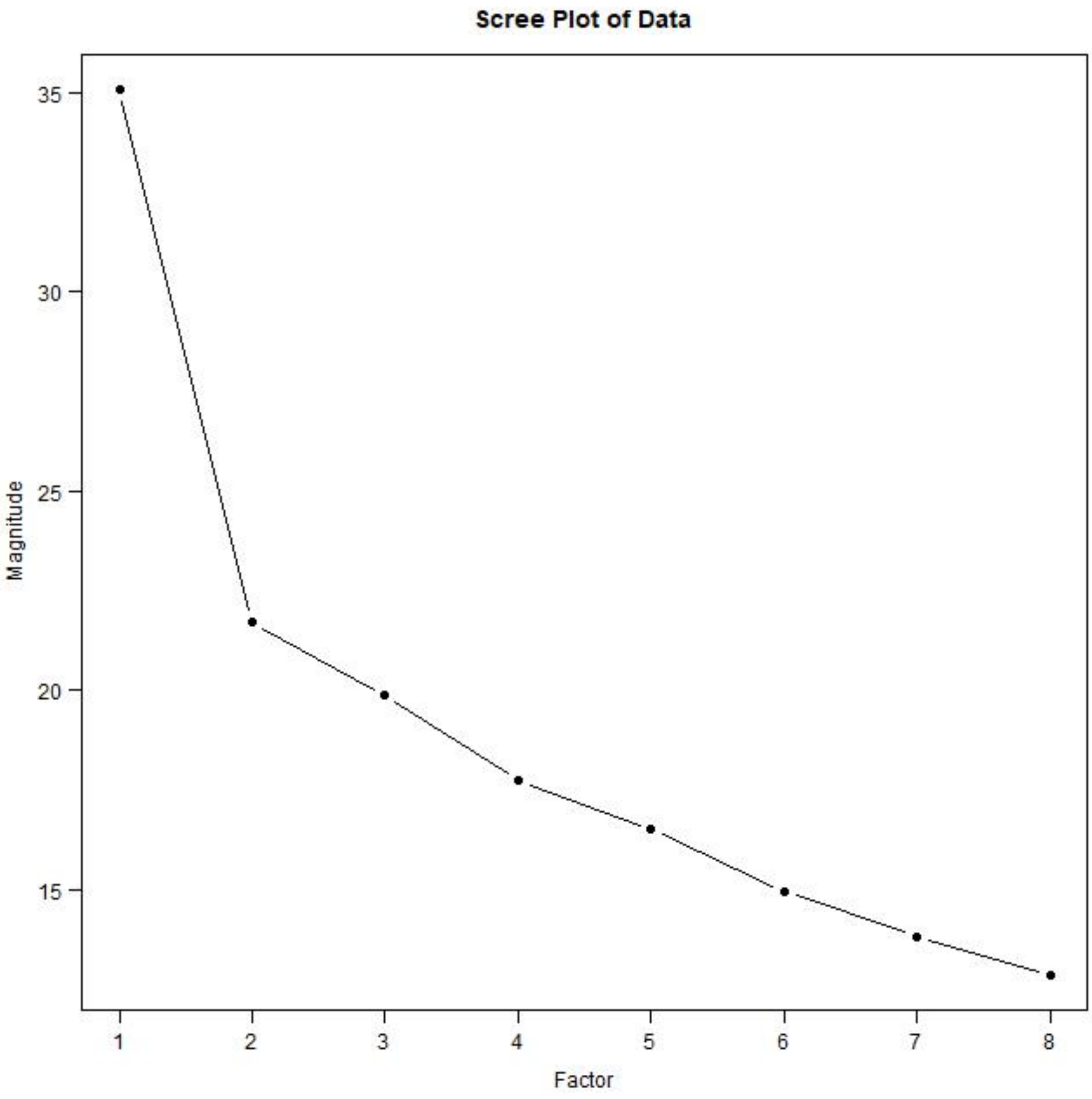

Figure S3: CCA scree plot after removing high-difficulty items with obscure truth values (A1, B2, B3, B4R), dichotomized following (M | O | A = 1, I | R | Q = 0), omitting four respondents with zero response variance.

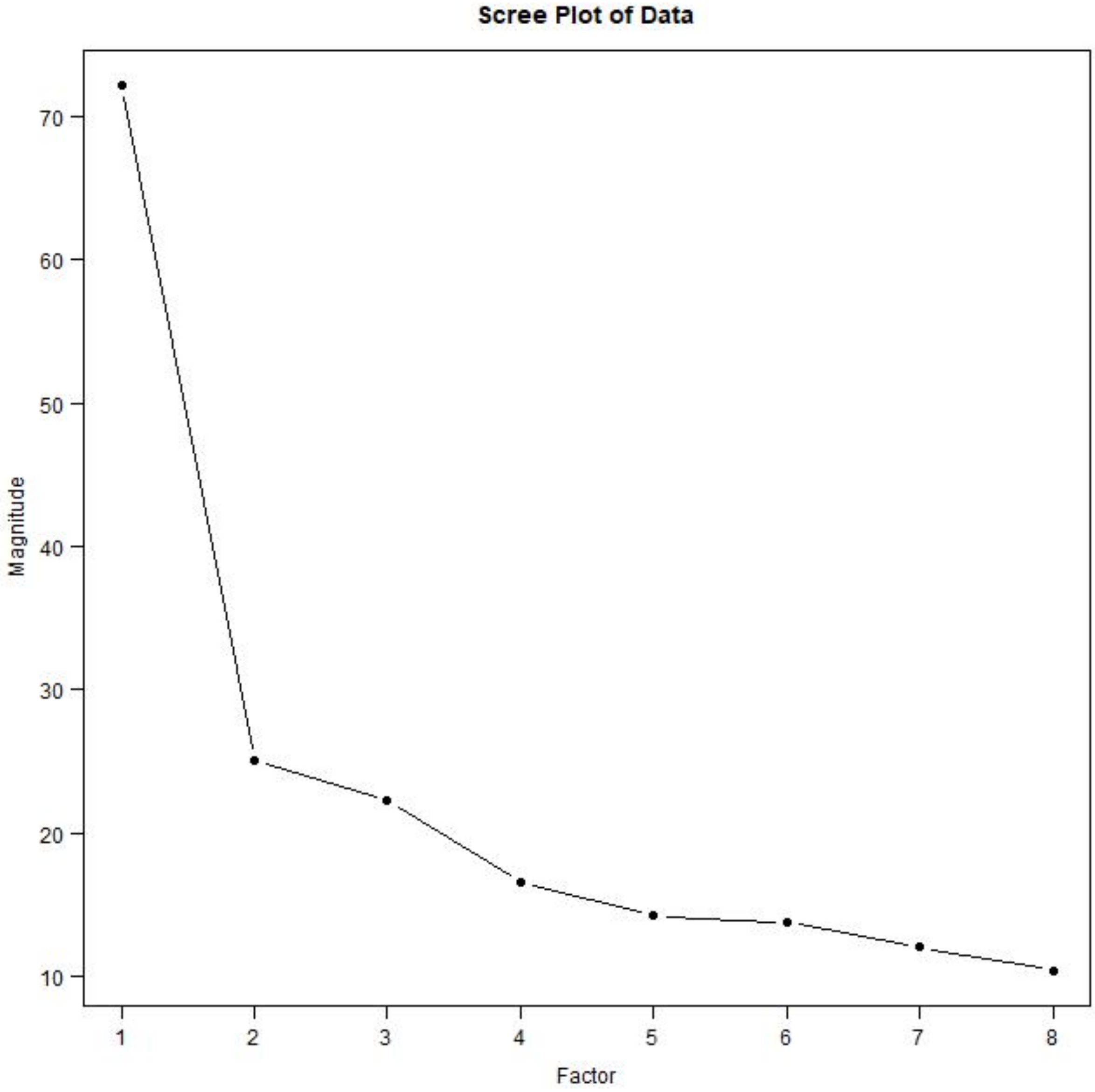

Supplement: 23-00199-Wachinger-Supplement.pdf [file 23-00199-Wachinger-Supplement.pdf]
